# MULTIMORBIDITY, MENTAL HEALTH, AND TERMINAL DECLINE IN LATER LIFE

**DOI:** 10.1093/geroni/igz038.2308

**Published:** 2019-11-08

**Authors:** Dorina Cadar, Lucy Stirland, Graciela Muniz Terrera

**Affiliations:** 1 University College London, London, United Kingdom; 2 University of Edinburgh, Centre for Clinical Brain Sciences, Edinburgh, United Kingdom; 3 University of Edinburgh, Edinburgh, United Kingdom

## Abstract

The close interlink between physical and mental health outcomes has long been recognised in gerontological research. Mental-physical comorbidities – the presence of at least one physical health long term condition, and at least one mental health-related long term condition are common in older age individuals. Numerous studies have shown a positive association between the prevalence of multimorbidity and age so, as the population of older individuals in developed nations continues to grow, multimorbidity is likely to become increasingly higher in ageing populations. A major goal in current gerontological neuropsychology and neuroepidemiological research is to better understand how interindividual differences in cognitive and mental health in old age emerge. Cognitive reserve (a marker of brain resilience) may come into play when facing stressors that affect cognitive decline and mental health, such as suffering from chronic diseases. We present data from three different longitudinal studies of ageing i) the Lothian Birth Cohort of 1921, ii) PREVENT and iii) the English Longitudinal Study of Ageing from the United Kingdom. These studies are ideally placed to address key research questions related to mental ageing, psychological health, terminal decline and their determinants. We explored the following objectives: 1) to investigate the association between an increasing number of chronic physical conditions, medication and mental disorders 2) to assess the role of childhood intelligence and education on the terminal decline in later life 3) to investigate the associations between different markers of cognitive reserve and dementia

